# Influence of Slide Burnishing Parameters on the Surface Layer Properties of Stainless Steel and Mean Positron Lifetime

**DOI:** 10.3390/ma15228131

**Published:** 2022-11-16

**Authors:** Agnieszka Skoczylas, Kazimierz Zaleski, Jakub Matuszak, Krzysztof Ciecieląg, Radosław Zaleski, Marek Gorgol

**Affiliations:** 1 Department of Production Engineering, Mechanical Engineering Faculty, Lublin University of Technology, 36 Nadbystrzycka, 20-618 Lublin, Poland; 2 Department of Materials Physics, Institute of Physics, Faculty of Mathematics, Physics and Computer Science, Maria Curie-Sklodowska University, Maria Curie-Sklodowskiej Sq. 5, 20-031 Lublin, Poland

**Keywords:** slide burnishing, X6CrNiTi18 stainless steel, surface roughness 3D, topography, microhardness, residual stress, positron mean lifetime *τ_mean_*

## Abstract

This paper presents the results of an experimental study on the impact of slide burnishing on surface roughness parameters (*Sa*, *Sz*, *Sp*, *Sv*, *Ssk*, and *Sku*), topography, surface layer microhardness, residual stress, and mean positron lifetime (*τ_mea_*_n_). In the study, specimens of X6CrNiTi18 stainless steel were subjected to slide burnishing. The experimental variables were feed and slide burnishing force. The slide burnishing process led to changes in the surface structure and residual stress distribution and increased the surface layer microhardness. After slide burnishing, the analyzed roughness parameters decreased compared with their pre-treatment (grinding) values. The slide burnishing of X6CrNiTi18 steel specimens increased their degree of strengthening *e* from 8.77% to 42.74%, while the hardened layer thickness *g_h_* increased after the treatment from about 10 µm to 100 µm. The maximum compressive residual stress was about 450 MPa, and the maximum depth of compressive residual stresses was *g_σ_* = 1.1 mm. The positron mean lifetime *τ_mean_* slightly yet systematically increased with the increase in burnishing force *F*, while an increase in feed led to changes of a different nature.

## 1. Introduction

The properties of the surface layer of machined elements have a significant influence on the service life of the components. In terms of service life, advantageous properties of the surface layer can be obtained by subjecting manufactured items to treatment methods such as burnishing [1], shot peening [2] and brushing [3]. Numerous studies have shown that the use of these treatment methods makes it possible to improve the functional properties of elements made of various materials. Following the use of shot peening, the fatigue life of AZ80 magnesium alloy was shown to increase by about 75% [4], while the burnishing of 41Cr4 steel resulted in an increase in its fatigue life by 22.7% [5]. The use of burnishing improved the fatigue life of X19NiCrMo4 steel shafts by 28.5% compared with non-burnished shafts [6]. The fatigue life of elements made of titanium alloys increased from 125 to 420% as a result of shot peening, depending on the process parameters such as impact energy and shot peening time [7]. Shot peening also affects the redistribution of the residual stress field in friction stir welding [8]. It was also found that burnishing led to an increased wear resistance of steel [9] and titanium alloy [10]. Comparative studies of surface wear after turning, grinding, ball burnishing, and vibroburnishing showed that the most wear-resistant surface was obtained after vibroburnishing [11]. Burnishing also reduces wear due to fretting [12].

The surface layer properties of elements subjected to burnishing and shot peening depend on many factors, including the structure of a burnishing tool [13], burnishing strategy [14], process parameters [15], pre-machining prior to burnishing [16], regularity of shot peening tool hits on the treated surface [17], and properties of the machining fluid [18]. Burnishing processes are analyzed using experimental and numerical methods [19]. An important aspect of the treatment carried out is the amount of energy consumed [20].

Depending on the impact of a burnishing tool on the burnished object, a distinction is made between roller burnishing [21], ball burnishing [16], and slide burnishing [18], which is a process wherein the tool works in sliding contact with the treated surface. The slide burnishing process can be carried out using tools with a polycrystalline diamond and cemented carbide tip [22]. The surface of the slide burnishing tool adjacent to the burnished surface usually has the shape of a spherical ball with a radius ranging from 1 to 4 mm, and the force with which the tool is pressed against the workpiece is usually in the range of 20–250 N [23,24,25]. Given the relatively low value of the tool force on the burnished element, the slide burnishing method can be used to shape the surface layer of objects with low stiffness. The advantage of slide burnishing is that its use causes a significant reduction in the surface roughness. For example, after the slide burnishing of carbon steel samples with an initial surface roughness of Ra = 1.39–12.90 µm, the surface roughness was reduced to Ra = 0.31–0.53 µm [26]. The authors of [27] stated that nano-crystalline structures in the 10–300 nm grain size range formed at the subsurface layer after slide burnishing normalized carbon steel. Grain sizes increase approximately linearly with the depth below the surface.

One of the types of elements subjected to burnishing and shot peening are made of stainless steels. These steels are used, among others, for the production of installation elements in the food, chemical, transport and paper industries, as well as parts of aircraft engines, automotive vehicles, and energy devices.

Increased fatigue life due to shot peening has also been observed for stainless steels. Research by Yang et al. showed that with an increase in the shot peening intensity of austenitic 304 stainless steel ranging from 0.1 mmA to 0.4 mmA, its compressive residual stresses and fatigue life increased [28]. A significant improvement in surface layer properties can be achieved by multiple shot peening. According to Chen et al., the use of three-time shot peening for SAF 2507 duplex stainless steel specimens with decreasing peening intensity allowed for an increase in the compressive residual stress, increase in the content of α’ martensite and reduce surface roughness compared with single-shot peening [29]. Menezes et al. obtained an increased depth of surface layer hardening as well as higher wear and corrosion resistance of AISI 316L stainless steel by using shot peening prior to the sequential plasma treatment of this particular steel grade [30]. Furthermore, the wear tests of 316L stainless steel carried out by Gopi et al. showed that shot peening reduced the mass wear and friction coefficient of samples made from this steel grade [31]. A study by Walczak et al. showed that shot peening conducted with the use of ceramic shots on 17-4PH stainless steel additively produced by direct metal laser sintering resulted in grain refinement, reduced surface roughness, as well as increased wear and corrosion resistance [32]. A study by Spadaro et al. showed that shot peening caused a greater increase in the fatigue life of 235MA austenitic stainless steel compared with laser shock processing [33].

Burnishing is also used as the finishing method for stainless steel components. Bouzid Sai et al. examined the surface layer of duplex stainless steel after turning, grinding, and burnishing with a 9 mm diameter ball, which showed that the best results were obtained after burnishing [34]. Attabi et al. reported the results of a study investigating the effect of the number of passes in the ball burnishing process for 316L stainless steel on the steel’s microhardness, nano-hardness, and wear resistance [35]. The wear resistance was also tested after the slide burnishing process. The authors of [36] proved that diamond burnishing CuAl8Fe3 aluminum bronze with six passes provided the highest wear resistance under the condition of boundary lubrication friction; the procedure also increased the wear resistance 5.1 times more than fine turning. Diamond burnishing with one pass resulted in the highest wear resistance under the dry friction condition and increased the wear resistance 1.75 times more than fine turning. Valiorgue et al. conducted a comparative study on the effect of turning, belt grinding, and roller burnishing on the properties of 15-5PH martensitic stainless steel, which demonstrated that the greatest compressive residual stress and highest fatigue life were obtained after the rolling burnishing process [37]. Konefal et al. investigated the corrosion resistance of X6CrNiMoTi17-12-2 stainless steel samples that were subjected to the following types of treatment: drawing, polishing, and slide diamond burnishing. The burnishing parameters were maintained at a constant *F* = 150 N force and *f* = 0.11 mm/rev feed. It was found that the burnished samples showed the greatest corrosion resistance [38].

The surface layer properties of elements can be tested with various techniques that use both destructive and non-destructive methods. Previous studies have shown that positron annihilation lifetime spectroscopy can be used to study the surface layer. This research technique allows for the estimation of the concentration of various material defects on an atomic scale (dislocations, vacancies, etc.), with these defects being positron trapping centers. In contrast to microscopic techniques, an advantage of this non-destructive method is the possibility of obtaining data that is averaged over a relatively large volume of the material (for steels, it is a layer with a thickness of several dozen micrometers on an area of about a square millimeter). Horodek et al. used positron annihilation methods to investigate the depth of a subsurface zone in 304 austenitic stainless steel processed by various processes such as laser cutting, abrasive water jet, and milling techniques [39]. Positron annihilation spectroscopy was also used to test 316L austenitic stainless steel that was subjected to laser shock peening [40]. Zaleski et al. [41] studied the influence of vibratory shot peening of C45 steel, 7075 aluminum alloy, and Ti6Al2Mo2Cr titanium alloy on the positron annihilation parameters. The influence of the impulse shot peening on the surface layer properties of Inconel 718 nickel alloy was also investigated using positron annihilation spectroscopy [42].

The literature review indicates that there is currently a lack of the studies on the surface layer properties of X6CrNiTi18 steel after a slide burnishing process has been carried out with annihilation techniques and simulation FEM. Therefore, it seems advisable to determine the impact of the technological parameters of slide burnishing on the surface layer properties with the use of positron annihilation and FEM analyses. The aim of this experimental and numerical study was to evaluate the effect of slide burnishing on the surface layer properties of X6CrNiTi18 stainless steel as well as on the positron lifetime.

## 2. Materials and Methods

Experiments were conducted on specimens of austenitic corrosion-resistant steel X6CrNiTi18 (1.4541) (in accordance with EN 10088-1: 2014). This steel grade is used in the chemical industry (for manufacturing equipment of plants producing nitric acid and its salts, chemical fertilizers). X6CrNiTi18 steel is also used to make transmission pipelines, heat exchangers, reactors (petrochemical industry), tanks, autoclaves, pasteurizers, mixers, pump elements, and cisterns (food industry). This corrosion-resistant steel grade is characterized by very good weldability regardless of the welding method used. X6CrNiTi18 steel is produced in the form of tubes, sheets (cold- and hot-rolled), bars, profiles, wires, flat-die and die forgings, and tapes [43].

Table 1 shows the chemical composition and selected properties of the tested material. The tests were carried out on the samples, which were shaped as thin-walled rings with the following dimensions: external diameter: d = 56 mm, internal diameter: d_o_ = 50 mm, and width: b = 10 mm.

The ring-shaped specimens were ground before the slide burnishing procedure. The machining process was carried out on a cylindrical grinder. The grinding process was conducted with a grinding wheel peripheral speed of *v_s_* = 30 m/s and a grinding depth of *a_p_* = 0.01 mm. An aloxite grinding wheel was used.

**Figure 1:**
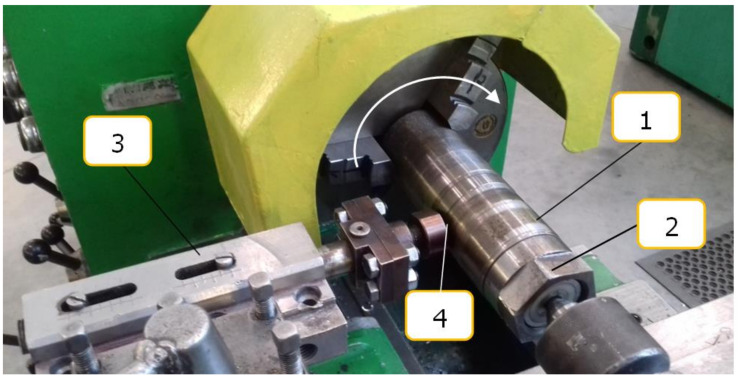
View of the test stand used in the experiment: 1: specimen; 2: mandrel with mounted samples; 3: slide burnishing tool; 4: diamond tip with radius R.

Slide burnishing was performed on a universal lathe (Figure 1). The test samples were mounted on a mandrel (2) that performed a rotary motion. A slide burnishing tool (3) with a spherical diamond tip (4) described by a radius R and burnishing force-exerting mechanism was pressed to the machined samples with a force F. The burnishing tool performed a feed motion f.

A tool with a spherical diamond tip with a radius of R = 3 mm was used for the slide burnishing process. Machine oil was used to reduce the friction during the slide burnishing. The oil used during slide burnishing was Mobil Vactra^TM^ Oil No. 2 synthetic oil (Mobil, Bavaria, Germany) The applied burnishing parameters selected based on preliminary tests and a literature review are listed in Table 2.

**Figure 2:**
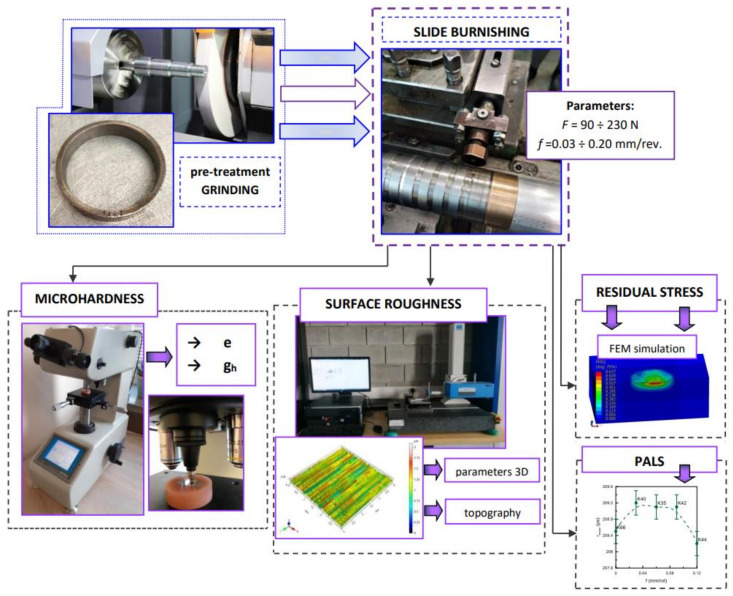
Methodology of the conducted research. The parameters of the experiment, stands research and achieved results.

In the next step, the selected properties of the surface layer of stainless steel after slide burnishing were examined, as shown in Figure 2.

The Hommel-Etamic T8000 RC120-400 device was used to measure the surface topography and surface roughness parameters in 3D. RC is a tactile roughness and contour measurement system. The scanned surface area was 3 × 3 mm. Measurements were carried out along the external surface of the samples (perpendicular to the traces after slide burnishing). The measurements were taken in compliance with the EN ISO 25178-2:2022 standard. The analyzed 3D parameters of the surface roughness were as follows:

*Sa*: arithmetical mean height of the surface;
*Sz*: maximum height of the surface;
*Sp*: maximum peak height of the surface;
*Sv*: maximum pit height of the surface;
*Ssk*: skewness;
*Sku*: kurtosis

The LM 700at microhardness tester (Leco, St. Joseph, MI, USA) was used to measure the surface layer microhardness before and after the slide burnishing process. The Vickers method was used for this purpose, which assumed an indentor weight of 50 g (HV 0.05). The specimens were subjected to standard treatment, and the angled microsections were examined. The results of the microhardness measurements were used to determine the degree of strengthening *e* and hardened layer thickness *g_h_*. Equation (1) was used to calculate the degree of strengthening.

(1) $$e=100\cdot \frac{{\mathrm{HV}}_{\max}-{\mathrm{HV}}_{o}}{{\mathrm{HV}}_{\max}},\;\%\;$$

where ${\mathrm{HV}}_{\max}$ is the maximum microhardness of the surface layer after slide burnishing and ${\mathrm{HV}}_{0}$ is the microhardness after grinding.

**Figure 3:**
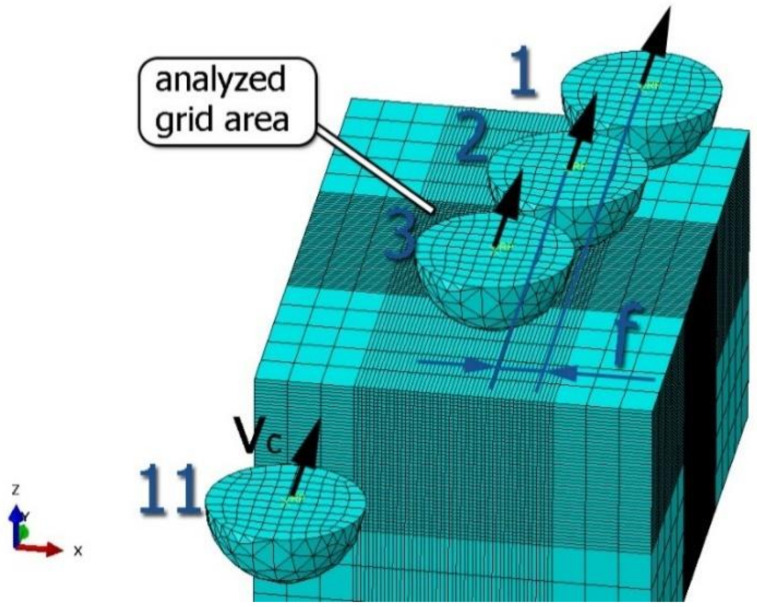
FEM simulation of the slide burnishing process.

The slide burnishing process was modelled using the explicit module of the Abaqus/CAE 2021 software. The simulations were carried out as part of dynamic calculations, considering the contact relations. The Johnson–Cook constitutive model was used with the following parameters: A = 280 MPa, B = 1215 MPa, n = 0.43, C = 0.031, and m = 1.15. The model considered the effect of strain hardening, strain rate, and temperature on the stress−strain relationship. The numerical model was modified in relation to the real one, yet it was ensured that all process conditions faithfully reproduced the real slide burnishing process. In the simulation, a 7.5 × 5 mm sample modelled with C3D8R-type elements was used, with the mesh density maintained in the central area of the burnished object. The grid size at the analysis site was 0.05 mm. The total number of elements in the mesh was 147,840, which amounted to 156,465 nodes. The burnishing element was modelled as a non-deformable solid using two types of finite elements: R3D4 (140 elements) and R3D3 (1840 elements), with compaction in the area of contact with the workpiece. The successive passes of the burnishing element in the real burnishing process were represented as hemispheres with a rounding radius equal to 3 mm. The distance of the burnishing elements used in the simulation was consistent with the feed per revolution. In the FEM simulation, 11 burnishing elements were used, which allowed for the burnishing process to be carried out on a length equal to 10 feed values per revolution of the burnishing element, which for the adopted process conditions gave a burnished surface length ranging from 0.3 to 2 mm. The area of the compacted mesh was 2.5 × 2.5 mm and was located in the center of the workpiece. In addition, along the Y-axis, each of the burnishing elements used in the simulation was offset from the next to represent the conditions in which the burnishing element would partially cover the previous pass of the tool after each revolution of the workpiece. This methodology made it possible so that in the case of a speed of *v_n_* = 35 m/min and simulation time of t = 0.008572 s, each of the burnishing elements covered a distance of 5 mm, thus burnishing the area with a dense mesh of the finite elements. The stresses S11 reflecting the residual stresses in the surface layer after the burnishing process and the PEEQ equivalent plastic strains were analyzed. Figure 3 shows the simulated burnishing process for the established 11 passes of the burnishing element, where the marked interval is equal to the feed (distance between successive burnishing element passes).

The non-zero equivalent plastic strain at variable feeds had a depth ranging from 0.4 mm (for *f* = 0.2 mm/rev) to 0.6 mm (for *f* = 0.03 mm/rev). In turn, during the experiment with variable burnishing force, the non-zero equivalent plastic strain had a depth ranging from 0.25 mm (for *F* = 90 N) to 0.8 mm (for *F* = 300 N).

**Figure 4:**
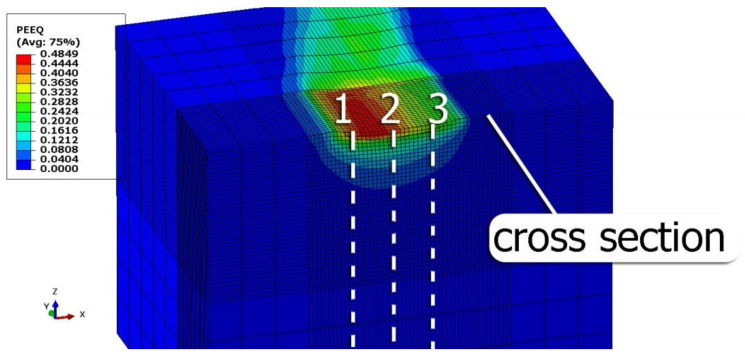
Method of creating paths for determining the residual stresses in the surface layer.

To evaluate the influence of the pressing force and feed rate on the effects of the process, FEM simulations were carried out for the parameters specified in Table 2. The S11 stress plots obtained from the simulations were determined as the average value of three cross-section lines perpendicular to the surface, as shown in Figure 4. The cross-sections were examined in the area of the compacted mesh under the symmetry axis of the burnishing element (taking into account the fixed feed per revolution in each case).

Positron annihilation lifetime spectra were measured with a digital lifetime spectrometer. Two scintillation detectors equipped with BaF_2_ scintillators fixed in the immediate vicinity of the samples were used to detect gamma quanta with an energy of 1274 keV (which indicates the formation of a positron in the β^+^ decay) and annihilation quanta with an energy of 511 keV. Voltage pulses from the detectors were recorded with the Agilent U1065A digitizer at a sampling rate of 4 GS/s and then examined with dedicated software [44] to determine the time difference between gamma and annihilation signals. ^22^NaCl (0.3 MBq) in an 8 µm-thick Kapton envelope served as a source of positrons and was placed between two identical samples fixed in a dedicated holder. The sample–source–sample “sandwich” was shifted below the edge of the scintillators to avoid the coincidence of the collinear annihilation quanta of 511 keV–511 keV. The total count number in the positron lifetime spectra slightly exceeded 2.1 × 10^7^ for each sample.

Results were analyzed using the PALSfit program [45], which allowed us to determine the lifetimes and relative intensities of the individual components as well as the positron mean lifetime. A good fit was obtained, assuming two dominant components of the lifetime spectrum. Typically for the low-background spectrum from a digital spectrometer, it was also necessary to assume a long-life component with a lifetime of approx. 1.5 ns and negligible intensity of ~0.2%. The contribution of a source correction (annihilation in the Kapton envelope) with a lifetime of 382 ps was determined at 14.3% using the Positron Fraction program [46]. A single Gaussian with an FWHM of 201 ps was sufficient to describe the resolution function.

## 3. Results and Discussion

The following subsections present the results of the research on the analyzed properties of the surface layer and lifetime of positrons.

### 3.1. Topography and Surface Roughness

**Figure 5:**
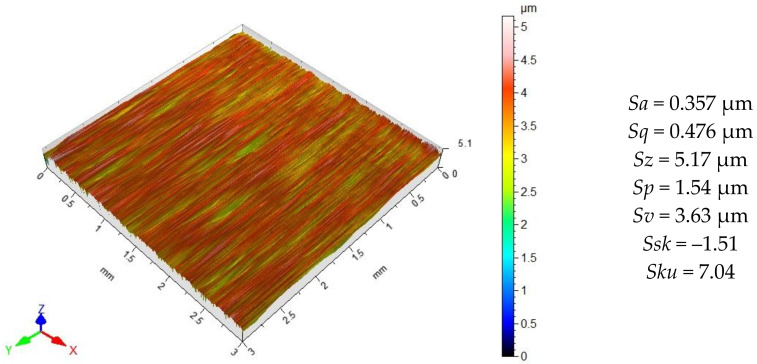
X6CrNiTi18 steel surface topography after grinding pre-treatment.

Micro-irregularities on the ground surface have a unidirectional pattern (Figure 5), which is characteristic of this machining method. There are numerous depressions on the surface, which are confirmed by the high value of the *Sv* parameter. The profile of the ground surface is characterized by sharp elevations and depressions that were formed on the surface as a result of the work by abrasive grains of the grinding wheel.

**Figure 6:**
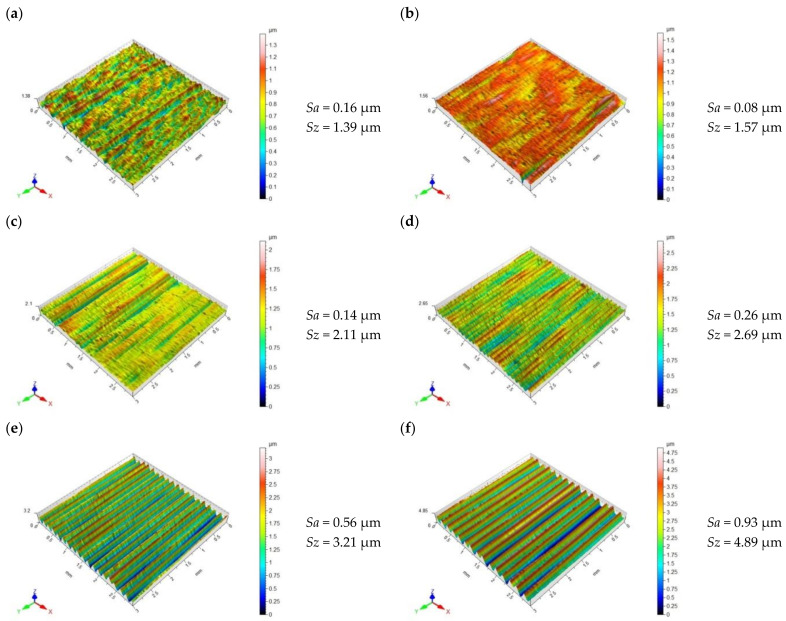
Topography of X6CrNiTi18 steel surface after slide burnishing: (**a**) *f* = 0.03 mm/rev., *F* = 230 N; (**b**) *f* = 0.06 mm/rev., *F* = 230 N; (**c**) *f* = 0.09 mm/rev., *F* = 230 N; (**d**) *f* = 0.12 mm/rev., *F* = 230 N; (**e**) *f* = 0.16 mm/rev., *F* = 230 N; (**f**) *f* = 0.20 mm/rev., *F* = 230 N.

After the slide burnishing process conducted with a feed *f* = 0.03–0.09 mm/rev (Figure 6a,c), the shape of the micro-irregularities was more irregular compared with the surface after grinding (Figure 5). There were numerous visible deformations of microinequalities that were not reflected in the burnishing kinematics. This is most likely due to the friction and adhesive interaction between the surface of the workpiece and the diamond tip of the tool. The use of the burnishing feed value of *f* = 0.12–0.20 mm/rev led to a deterioration of the geometric structure of the surface. For *f* = 0.12–0.20 mm/rev (Figure 6d,e), a characteristic unidirectional pattern of surface micro-irregularities was visible, with noticeable elevations and depressions on the surface. The obtained values of the *Sa* parameter were greater than the *Sa* values after grinding. For the entire tested feed range, the obtained values of the *Sz* parameter were lower than those after the pre-treatment. The minimum values of *Sa* and *Sz* were obtained for *f* = 0.06 mm/rev.

**Figure 7:**
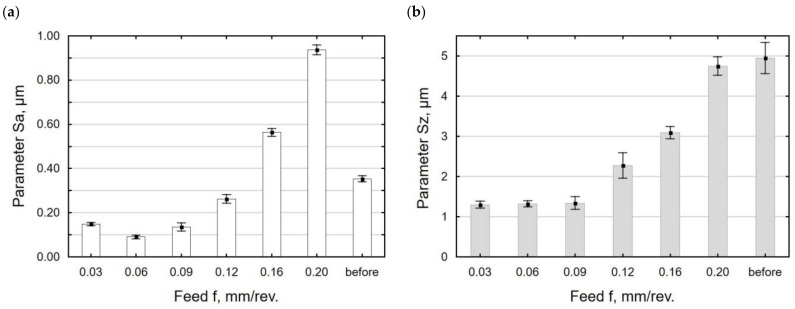
Feed versus surface roughness parameters *Sa* (**a**) and *Sz* (**b**) (*F* = const. = 230 N, *v_n_* = 35 m/min, *i* = 1).

**Figure 8:**
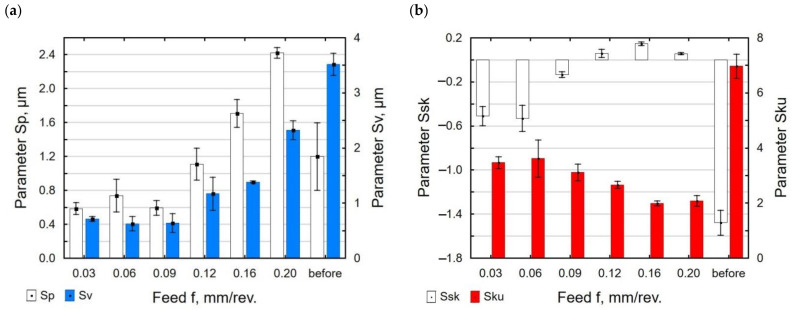
Feed versus surface roughness parameters: *Sp* and *Sv* (**a**); *Ssk* and *Sku* (**b**) (*F* = const. = 230 N, *v_n_* = 35 m/min, *i* = 1).

Figure 7 and Figure 8 present the influence of the feed value on the analyzed 3D surface roughness parameters. It can be seen that for the burnishing feeds in the lower range, the analyzed surface roughness parameters decreased to the minimum for *f* = 0.06 mm/rev.; a further increase in the burnishing feed led to an increase in the surface roughness. The increase in the burnishing feed increased the distance between successive passes of the tool (no deformation of surface micro-irregularities after the pre-treatment), which caused an increase in the *Sa*, *Sz, Sp*, and *Sv* parameters. The increase in the *Sa* parameter for *f* = 0.03 mm/rev. may be due to multiple deformations of the same surface micro-irregularities. For the feed ranging *f* = 0.03–0.09 mm/rev. (Figure 7b), no changes in the *Sz* parameter value can be observed. Similar changes in the roughness parameters as a function of the feed in the slide burnishing of AISI 316Ti steel were observed by Maximov et al. [47]. After the slide burnishing of X6CrNiTi18 steel, the surface roughness profile is changed. In the total surface profile after slide burnishing, the elevations (parameter *Sp*) had a greater share than the depression (parameter *Sv*). After grinding, the surface micro-irregularities were deformed, and their shape and dimensions were changed. The values of the skewness ratio *Ssk* and kurtosis *Sku* were changed as well. For *f* = 0.12 mm/rev. the obtained values were *Ssk* = 0.058 and *Sku* = 2.66, which means that there will be less friction between the mating surfaces, which agrees with the results reported in [48]. The same properties will be characteristic of the surface after grinding [49], but with a significantly worse surface quality (high values of the *Sa* and *Sz* parameters).

**Figure 9:**
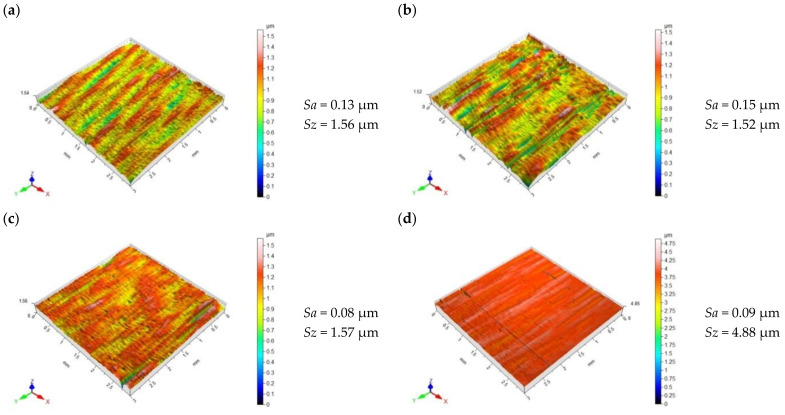
Topography of X6CrNiTi18 steel surface after slide burnishing (**a**) *F* = 90 N, *f* = 0.06 mm/rev.; (**b**) *F* = 160 N, *f* = 0.06 mm/rev.; (**c**) *F* = 230 N, *f* = 0.06 mm/rev.; (**d**) *F* = 300 N, *f* = 0.06 mm/rev.

**Figure 10:**
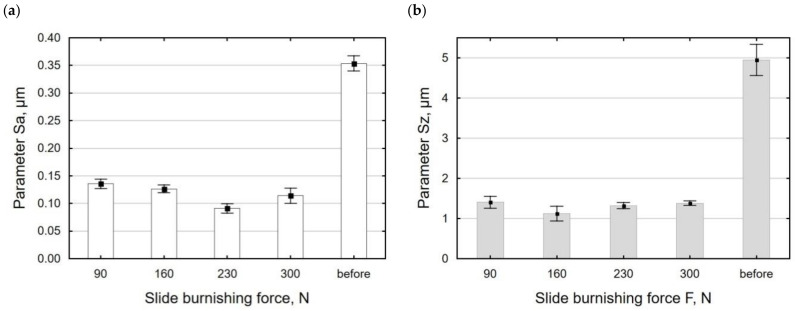
Burnishing force versus surface roughness parameters *Sa* (**a**) and *Sz* (**b**) (*f* = const = 0.06 mm/rev., *v_n_* = 35 m/min, *i* = 1).

Figure 9 shows the surface topography of X6CrNiTi18 steel after the slide burnishing process when conducted with a different force value. The use of a higher force value caused the deformation of the sample surface to be “fuller”; the surface micro-irregularities were smoothed after the pre-treatment, which translated into a four-fold reduction in the *Sa* parameter for *F* = 230 N. The surface topography obtained after slide burnishing when conducted with *F* = 90–230 N was characterized by flattened micro-irregularities. The obtained values of the parameter *Sz* for the force *F* = 90–230 N had similar values. The use of the force *F* > 230 N caused a slight increase in the *Sa* and *Sz* parameters (Figure 10); the “flattening” of the surface and the presence of individual surface defects were also visible. The presence of these surface defects leads to a higher *Sz* value. It should be explained that the use of higher burnishing forces causes shear damage under the sample surface and surface flaking [50]. The obtained changes in the *Sa* and *Sz* parameters as a function of the force *F* are similar to the results described in [49,50]. The surface roughness parameters *Sa* and *Sz* ranged from 62% to 77% lower compared with the values obtained after grinding.

**Figure 11:**
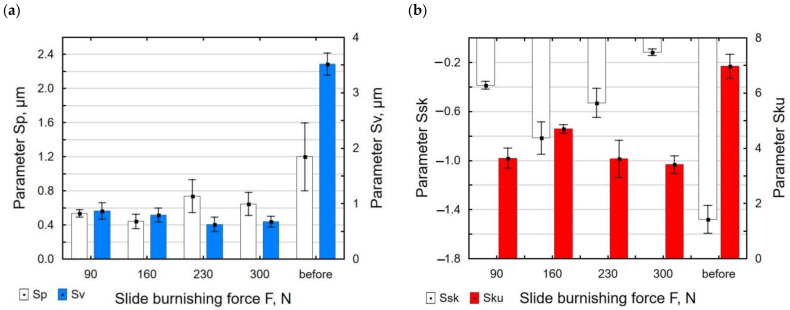
Burnishing force versus surface roughness parameters: *Sp* and *Sv* (**a**); *Ssk* and *Sku* (**b**) (*f* = const. = 0.06 mm/rev., *v_n_* = 35 m/min, *i* = 1).

During slide burnishing, the diamond tip is in constant contact with the surface of the workpiece. This causes intensive deformation of the surface micro-irregularities. As a result, the values of the *Sp* and *Sv* parameters decreased from 36% to 82% compared with their values after grinding (Figure 11a). The kurtosis coefficient *Sku* and asymmetry *Ssk* also changed (Figure 11b). The obtained absolute values of the *Ssk* and *Sku* coefficients were smaller than those after grinding, but the values of *Ssk* < 0 and *Sku* > 0 mean that the material was concentrated around the profile peaks; therefore, this surface can be considered to be a good bearing surface [49].

### 3.2. Microhardness

**Figure 12:**
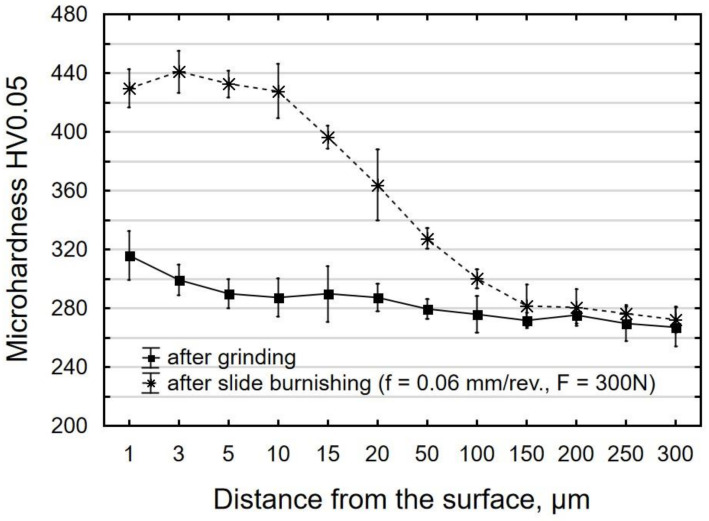
Distribution of microhardness on the surface layer of samples after grinding (pre-treatment) and slide burnishing.

As a result of slide burnishing and grinding, the material was strengthened (Figure 12). Following grinding conducted close to the surface at a depth of 1 µm, the microhardness of the surface layer was approx. 15% higher than the microhardness of the core. From a depth of 5 µm, the microhardness of the surface layer of the ground sample was similar to the microhardness of the core. The distribution of microhardness for the slide burnished sample shows that the highest microhardness occurred at a depth of 3 µm from the surface. The obtained microhardness distribution is characteristic of this treatment method.

**Figure 13:**
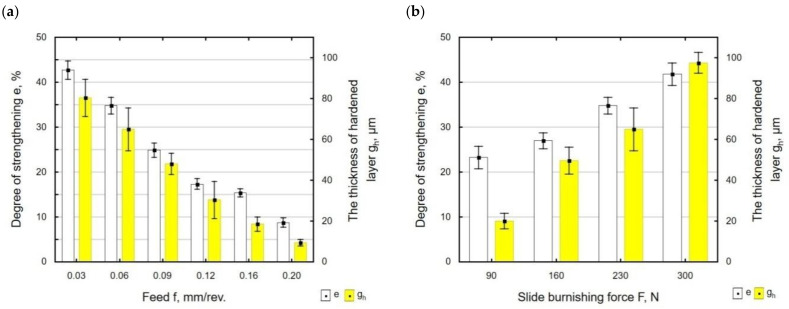
Degree of strengthening and hardened layer thickness after slide burnishing as a function of: (**a**) feed *f* (*F* = 230 N, *v_n_* = 35 m/min, *i* = 1); (**b**) force *F* (*f* = 0.06 mm/rev., v*_n_* = 35 m/min, *i* = 1).

Figure 13 shows the degree of strengthening *e* and hardened layer thickness *g_h_* as a function of burnishing feed (Figure 13a) and slide burnishing force (Figure 13b). The hardening degree was determined for the depth *g_h_* = 3 µm, for which the microhardness is the highest. The slide burnishing of X6CrNiTi18 steel specimens resulted in the degree of strengthening *e* ranging from 8.77% to 42.74%, which was higher than the degree of strengthening obtained for X19NiCrMo4 steel (*e* = 32%) [6]. The hardened layer thickness *g_h_* after slide burnishing ranged from about 10 µm to 100 µm, while in [6], the reported changes in microhardness reached a depth of up to 18 μm.

When burnishing is conducted with a higher feed (Figure 13a), the traces of the diamond tip passes are at a greater distance from each other. This causes a decrease in the structural homogeneity and, consequently, a lower degree of strengthening *e* and decrease in the hardened layer thickness *g_h_*.

The greatest differences between the values of the strengthening degree *e* occurred for the forces *F* = 230 N and *F* = 300 N. The application of a higher burnishing force caused an increase in the degree of strengthening *e*. This is most likely due to intense plastic deformation caused by friction, which leads to grain refinement of the microstructure. The use of a higher burnishing force causes the plastic deformation to take place deeper in the material, which results in increased microhardness of the surface layer extending further from the treated surface. The obtained results of the influence of *F* on *g_h_* are similar to the results described in [26], where the slide burnishing process was conducted on carbon steel with a hardness of 250 HV.

### 3.3. Residual Stress

**Figure 14:**
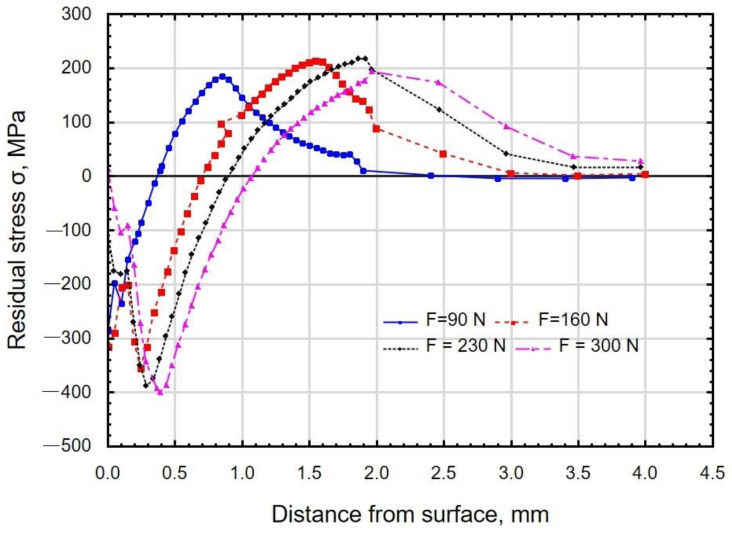
Distribution of residual stresses as a function of distance from the surface of X6CrNiTi18 steel samples after slide burnishing with a variable burnishing force (*f* = 0.06 mm/rev; *v_n_* = 35 m/min; *i* = 1; R = 3 mm).

Figure 14 shows the results of the influence of the burnishing force on the distribution of stresses S11 occurring in the surface layer. As the burnishing force increased, the depth of compressive stresses increased. The maximum value of the compressive residual stress (about 400 MPa) was obtained for the burnishing forces *F* = 230 N and *F* = 300 N. The burnishing force significantly affected the depth of compressive residual stresses from *g_σ_* = 0.4 mm (for the force *F* = 90 N) to *g_σ_* = 1.1 mm (for the force *F* = 300 N). It should be assumed that the increase in the depth of residual stresses will allow the place of fatigue crack initiation to be shifted from the surface to the subsurface layers. This means that the phenomenon of nucleation and crack propagation will be delayed [51].

**Figure 15:**
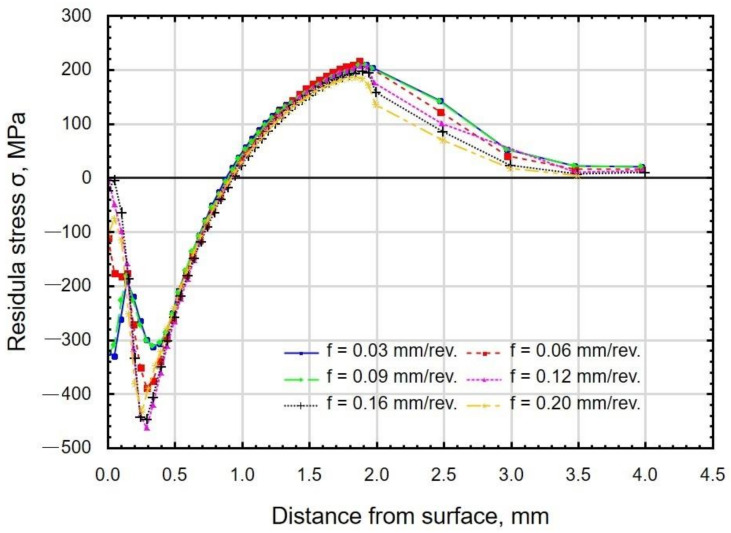
Distribution of residual stresses as a function of distance from the surface of X6CrNiTi18 steel samples after slide burnishing with a variable feed (*F* = 230 N; *v_n_*= 35 m/min; *i* = 1; R = 3 mm).

Figure 15 shows the effect of burnishing feed on the distribution of S11 stresses. The depth of the compressive residual stresses was about *g_σ_* = 0.9 mm. No significant influence of the feed on the residual stress distribution was observed. The obtained depths of residual stresses are greater than those observed after the slide burnishing of C45 steel [52]. The compressive residual stress zone was much deeper than the plastically deformed zone. The results are similar to those presented in the paper [53].

Table 3 shows the visualization of the PEEQ equivalent plastic strains. For variable burnishing forces, the feed rate *f* = 0.06 mm/rev. was used; for variable feeds, the burnishing force was *F* = 230 N. A symmetrical cross-section was created to illustrate deformation on the surface as well as in the subsurface layers. The PEEQ color maps demonstrate that the plastic deformation wave resulting from the passages of successive burnishing elements shifted the area of maximum plastic deformation concentration away from the axis of symmetry of the workpiece. This phenomenon was not observed for the feeds *f* = 0.16 mm/rev. and *f* = 0.20 mm/rev.

### 3.4. Positron Annihilation Lifetime Spectroscopy

The obtained positron lifetimes and intensities of both components in individual samples slightly differed from each other. The short-lived component with an intensity of 80.5 ± 0.3% dominated in the spectra, and its lifetime of 168.7 ± 0.3 ps indicated its origin to mainly come from the positrons trapped at edge dislocations, where a lifetime of 162 was observed [54]. The lifetime of the second component was 360 ± 2 ps and corresponds to quite large vacancy clusters, i.e., approx. 15 vacancies [55]. However, it should be noted that this is an average value and that the vacancies can vary in size. Due to slight changes in lifetimes and intensities, the differences between the samples are best reflected by a change in the mean lifetime (*τ_mean_*), defined as:

(2) $$\tau_{\mathrm{mean}}=\frac{\tau_{1}I_{1}+\tau_{2}I_{2}}{I_{1}+I_{2}}$$

where τ_1_, τ_2_ and *I*_1_, *I*_2_ are the lifetimes and the intensities of the first and second components of the positron lifetime spectrum, respectively. An increase in *τ_mean_* means an increase in the probability of positron trapping in vacancy clusters (most likely due to an increase in their concentration) and/or an increase in the average size of the clusters. It has been shown that this parameter is a good indicator of changes in the microhardness of the surface layer of samples [42].

**Figure 16:**
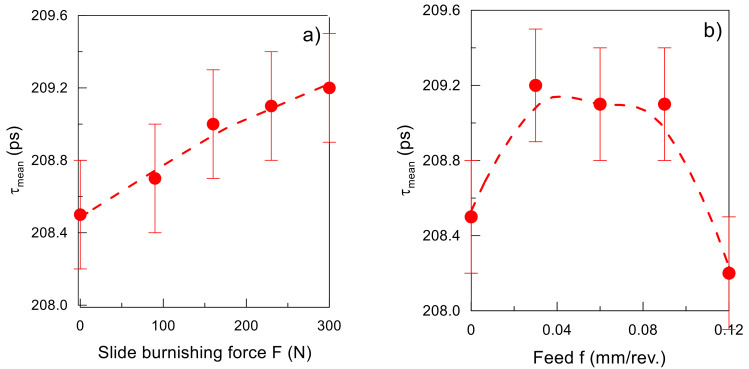
Mean positron lifetime *τ_mean_* as a function of (**a**) burnishing force *F* (*f* = 0.06 mm/rev., *v_n_* = 35 m/min, *i* = 1), (**b**) burnishing feed *f* (*F* = 230 N, *v_n_* = 35 m/min, *i* = 1). The dashed lines are only an eye-guide.

The positron mean lifetime *τ_mean_* increased slightly yet systematically with an increase in the burnishing force *F* (Figure 16). This dependence is consistent with the results of the microhardness measurements (Figure 12 and Figure 13). On the other hand, the decreasing dependence of microhardness on burnishing feed *f* was not reproduced by the *τ_mean_*. This suggests a different nature of the change in material defects when changing burnishing feeds than those induced by changing the burnishing force. The correlation of the dependence between *τ_mean_* and burnishing feed with an analogous dependence for the *Sa* parameter suggests that the change in surface topography and roughness may also affect the positron implantation profile.

## 4. Conclusions

Based on the results of slide burnishing of X6CrNiTi18 steel specimens conducted with variable technological parameters, the following conclusions can be drawn:

1. The use of the burnishing feed ranging *f* = 0.03–0.12 mm/rev. and slide burnishing force ranging *F* = 90–300 N led to a decrease in the analyzed surface roughness parameters (*Sa*, *Sz*, *Sp*, and *Sv*) in relation to their reference values (after grinding).
2. The surface shape after slide burnishing was plastically deformed. Changes in the shape and dimensions of the surface micro-irregularities were visible.
3. The obtained skewness value and kurtosis for the surface after slide burnishing mean that this surface can be considered a good bearing surface.
4. After slide burnishing, the surface layer was strengthened. The microhardness of the surface layer and depth of the hardened layer *g_h_* increased. The maximum degree of strengthening *e* was obtained after slide burnishing conducted with *F* = 230 N and *f* = 0.03 mm/rev (*e* = 42%), while the greatest increase in the hardened layer thickness of *g_h_* = 97.50 μm was obtained for *F* = 300 N and *f* = 0.06 mm/rev.
5. The FEM simulations of the slide burnishing process demonstrated the favorable nature of the residual compressive stresses occurring in the subsurface zones. It was noticed that the slide burnishing force had a greater impact on the depth of the compressive residual stresses than the feed.
6. The plastic deformation wave moved the area of concentration of the maximum equivalent plastic strains from the axis of symmetry of the workpiece toward the end passes of the burnishing element.
7. The positron mean lifetime *τ_mean_* increased slightly yet systematically with the increase in the burnishing force *F*; with an increase in feed, those changes had a different nature.

## Figures and Tables

**Table 1:** Chemical composition and selected properties of X6CrNiTi18 stainless steel.

Element	Fe	Cr	Ni	Mn	Si	P	S
wt%	70.4	18.9	8.3	1.7	0.5	0.1	0.1
Yield point (min)				220 MPa			
Tensile strength (max)				720 MPa			
Hardness (max)				215 HB			

**Table 2:** Technological parameters of slide burnishing (F: slide burnishing force; f: feed, *v_n_*: burnishing speed; *i*: number of passes).

No.	*F* [N]	*f* [mm/rev]	*v_n_* [m/min]	*i*
1.	90	0.06	35	1
2.	160			
3.	230			
4.	300			
5.	230	0.03		
6.		0.09		
7.		0.12		
8.		0.16		
9.		0.20		

**Table 3:** Influence of burnishing force and feed on equivalent plastic strains.

Burnishing Force (N) (*f* = const. = 0.06 mm/rev.)		Burnishing Feed (mm/rev) (*F* = const = 230 N)	
90	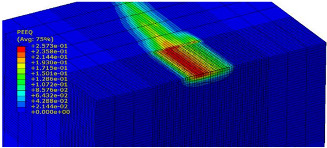	0.03	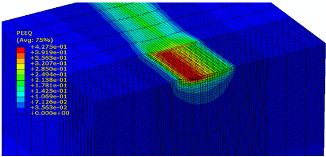
160	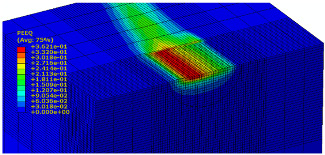	0.06	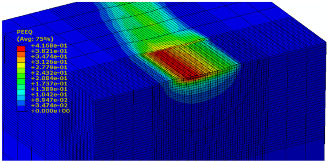
230	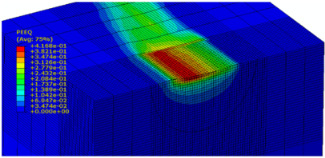	0.09	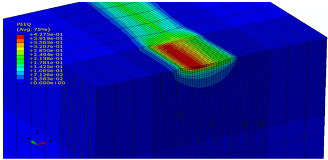
300	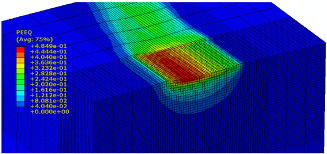	0.12	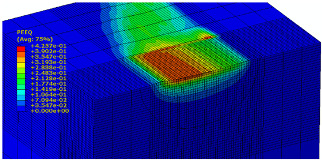
		0.16	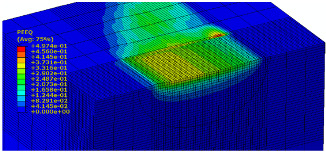
		0.20	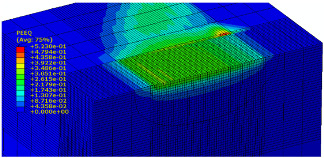

## Data Availability

The data presented in this study are available on request from the corresponding author.
